# Emerging Resistance and Shifting Bacterial Profiles in Lower Respiratory Tract Infections: A Tertiary Care Perspective

**DOI:** 10.7759/cureus.114836

**Published:** 2026-08-20

**Authors:** Firoz B Shaik, Ravindra V Shinde, Satish R Patil

**Affiliations:** 1 Department of Microbiology, Krishna Institute of Medical Sciences, Krishna Vishwa Vidyapeeth (Deemed to be University), Karad, IND

**Keywords:** bacteria resistant to multiple drugs, gram-negative bacteria, infection of the lower respiratory tract, resistance to antimicrobials, testing for susceptibility to antimicrobials

## Abstract

Background and objective

Lower respiratory tract infections (LRTIs) continue to pose a significant global public health concern, with bacterial agents responsible for a considerable proportion of cases requiring hospitalization and antibiotic treatment. The increasing emergence of antimicrobial resistance (AMR) among respiratory pathogens further complicates disease management and clinical outcomes. This study aimed to identify the bacterial profile of clinical LRTI cases and to determine the antimicrobial susceptibility of the identified pathogens.

Materials and methods

The study involved laboratory-based investigation of bacterial isolates from hospitalized patients with clinical suspicion of LRTI at Krishna Hospital and Medical Research Center, Karad, India, over one year from May 2024 to May 2025. Clinical specimens, such as endotracheal aspirates, sputum, and pleural fluid samples, were processed according to standard microbiological protocols.

Results

The study included 120 cases of confirmed LRTI. The 20-29 years age category exhibited the greatest infection burden, representing 35 (29.17%) cases, while the 30-39 years age group followed with 26 (21.67%) cases. Males were more frequently impacted than females, accounting for 77 (64.17%) cases versus 43 (35.83%). Of the 120 bacterial isolates obtained, *Pseudomonas *spp. were the predominant organism isolated, representing 49 (40.83%) of the isolates, followed by *Acinetobacter *spp. (n = 39, 32.50%) and *Klebsiella *species (n = 16, 13.33%),* Escherichia coli (E. coli)* (6, 5.00%), *Streptococcus *spp. (n = 5, 4.17%) and *Staphylococcus aureus (S. aureus)* ( n = 5, 4.17%). Endotracheal tube (ETT) specimens were the main source of isolates (n = 65, 54.17%), followed by sputum specimens (n = 49, 40.83%) and pleural fluid specimens (n = 6, 5.00%). Antimicrobial susceptibility testing (AST) revealed varied susceptibility patterns, with significant resistance noted among the isolates, especially in Gram-negative isolates. Of the 110 Gram-negative isolates, 96 were recognized as multidrug-resistant (MDR).

Conclusions

This investigation showed that Gram-negative bacterial pathogens dominate LRTI, with* Pseudomonas *spp.and* Acinetobacter *spp.* *being the most commonly isolated species. Most bacterial isolates were recovered from ETT and sputum specimens, underscoring the significance of appropriate diagnostic specimens in the diagnosis of LRTIs. The widespread occurrence of MDR Gram-negative isolates and the significant resistance to commonly used antimicrobial agents highlight the growing challenge of AMR. These results underscore the significance of prompt microbiological diagnosis, regular culture and AST, susceptibility-guided therapy, effective infection control measures, and antimicrobial stewardship initiatives to improve patient outcomes and reduce the transmission of resistant pathogens.

## Introduction

Lower respiratory tract infections (LRTIs) remain a major cause of morbidity and mortality worldwide, particularly in developing countries, and are frequently associated with Gram-negative bacterial pathogens. LRTIs encompass infections affecting the lungs, bronchi, bronchioles, and trachea, including acute bronchitis, bronchiolitis, and pneumonia. Global estimates indicate that respiratory tract infections continue to account for a significant share of hospital admissions and deaths related to infectious diseases, particularly among children, the elderly, and immunocompromised individuals [1,2]. The impact of LRTIs is exacerbated by factors such as population expansion, urban development, air pollution, tobacco use, chronic respiratory illnesses, and the rising prevalence of antimicrobial resistance (AMR) [2,3].

Numerous microorganisms, such as bacteria, viruses, and fungi, cause infections in the lower respiratory tract. Both Gram-positive and Gram-negative bacteria have been implicated in LRTIs; however, Gram-negative bacilli have emerged as important contributors to respiratory infections, particularly among hospitalized patients and those requiring intensive medical care [4]. Microorganisms like *Pseudomonas aeruginosa (P. aeruginosa), Klebsiella pneumoniae (K. pneumoniae), Acinetobacter *spp.*, *and *Escherichia coli (E. coli)* are commonly associated with serious respiratory infections and are increasingly noted for their capacity to acquire resistance to various antimicrobial agents [5,6]. These pathogens have various virulence factors, such as biofilm formation, toxin production, and the ability to persist in medical environments, all of which increase their toxicity and persistence.

The emergence and rapid spread of AMR among respiratory pathogens has become a significant global public health issue. AMR reduces the efficacy of widely used antibiotics, resulting in treatment failure, extended hospital stays, elevated healthcare costs, and increased mortality rates [2,7]. A major concern is the rise of multidrug-resistant (MDR) bacteria, which are resistant to several classes of antimicrobial agents and greatly restrict treatment options. The misuse of antibiotics, self-medication, inadequate infection control practices, and selective pressure from antimicrobial use have all contributed to the rising prevalence of multidrug-resistant organisms globally [7,8]. Recent research has highlighted an increasing prevalence of MDR Gram-negative bacteria in respiratory infections, underscoring the necessity for ongoing surveillance and effective antimicrobial stewardship initiatives [5,9].

Identifying the bacterial pathogens responsible for infections and assessing their AMR patterns is essential for the effective management of LRTIs. Culture-based diagnosis and antimicrobial susceptibility testing (AST) provide important insights into the local epidemiology of bacterial pathogens and assist clinicians in choosing appropriate antimicrobial therapy [4,10]. Understanding regional resistance patterns is crucial for guiding empirical therapy, avoiding antibiotic misuse, and reducing the emergence of resistant strains. Additionally, consistent monitoring of isolated bacteria and their resistance patterns can aid in developing evidence-based treatment protocols and infection control measures.

Given the rising incidence of bacterial respiratory infections and the growing challenge of AMR, the current study was conducted to identify the bacterial profile of clinical cases of LRTIs and to determine the antimicrobial susceptibility of the identified pathogens. The study also sought to evaluate the prevalence of multidrug-resistant bacterial isolates in patients attending a tertiary care hospital. The findings may provide important insights into local bacterial epidemiology and support the development of improved strategies for antimicrobial therapy and infection control.

## Materials and methods

Study design and setting

This was a hospital-based cross-sectional observational study conducted in the Department of Microbiology, Krishna Institute of Medical Sciences, Krishna Vishwa Vidyapeeth (Deemed to be University), and Krishna Hospital and Medical Research Centre, Karad, Maharashtra, India, from May 2024 to May 2025.

Sampling technique

Consecutive sampling was used. All eligible bacterial isolates recovered from clinically diagnosed LRTI cases during the study period were included consecutively. Consecutive sampling was chosen to minimize investigator discretion and reduce selection bias that may occur with convenience sampling, as all eligible cases presenting during the predefined study period had an opportunity to be included. This approach also reduces the likelihood of selectively enrolling participants based on specific demographic or clinical characteristics. Nevertheless, consecutive sampling cannot completely eliminate selection or referral bias, particularly in a single-center hospital-based study, and therefore the findings may have limited generalizability to other healthcare settings or the broader community.

Sample Size

The sample size was calculated using a previously reported prevalence of 38% from the study by Syed Mustaq Ahmed et al. [11]. The required sample size was determined using the following formula:

$\displaystyle{\displaylines{n = 4pq/L&sup2;}}$

Where: p = prevalence (38), q = 100 − p = 62, L = allowable error (10%), n = (4 × 38 × 62)/(10²), and n = 94.24 ≈ 95

Thus, using the prevalence reported by Syed Mustaq Ahmed et al. [11], the minimum required sample size was calculated to be 95. During the study period, 1,125 clinical specimens were processed, of which 652 showed microbial growth. Of these culture-positive specimens, 120 bacterial isolates recovered from clinically diagnosed LRTI cases fulfilled the inclusion criteria and were included in the final analysis.

Inclusion and exclusion criteria

Bacterial isolates recovered from clinical specimens obtained from patients with clinically diagnosed LRTIs, including sputum, endotracheal aspirates, and pleural fluid samples, from individuals of all age groups and both sexes, were included in the study. Viral and fungal pathogens, duplicate isolates from the same patient, and contaminated specimens were excluded from the analysis.

Specimen collection and processing

Following approval from the Institutional Ethics Committee of Krishna Vishwa Vidyapeeth (Deemed to be University) (Approval No. KVV/IEC/05/2024), the study was initiated. A total of 120 clinical specimens obtained from patients with clinically confirmed LRTIs were analyzed. Patients presenting to Krishna Hospital for the management of LRTIs were included in the study, and all participants provided informed consent. Clinical samples, such as sputum, endotracheal aspirates, and pleural fluid, were collected aseptically from patients of various ages and both sexes. All samples were collected in sterile containers and processed according to standard microbiological protocols. Direct smears were prepared from each specimen and subjected to Gram staining for initial microscopic examination. The stained smears were examined under oil immersion, and the morphological characteristics of the organisms, including Gram reaction, size, shape, and arrangement, were noted. The samples were subsequently cultured for bacterial isolation and identification, AST, and assessment of MDR bacterial isolates using standard microbiological techniques.

Isolation and identification of bacterial strains

The clinical specimens were inoculated onto MacConkey agar, blood agar, and chocolate agar and incubated under aerobic conditions at 37 °C for 18-24 hours. After incubation, the cultures were examined for bacterial growth, and individual colonies were subjected to Gram staining for initial identification. Bacterial isolates were identified using conventional microbiological methods based on colony morphology, Gram staining, and appropriate biochemical tests.

Antimicrobial susceptibility testing

AST was performed manually using the Kirby-Bauer disk diffusion method. The susceptibility findings were interpreted according to the Clinical and Laboratory Standards Institute (CLSI) 2024 guidelines and categorized as susceptible, intermediate, or resistant. Quality control of antimicrobial susceptibility testing was performed using standard reference strains, including *Escherichia coli* ATCC 25922, *Pseudomonas aeruginosa* ATCC 27853, and *Staphylococcus aureus* ATCC 25923, in accordance with CLSI recommendations. Bacterial isolates were categorized according to the number of antimicrobial classes to which they exhibited resistance. Isolates resistant to at least one antimicrobial agent in one antimicrobial class were categorized as R1, while isolates resistant to at least one antimicrobial agent in two antimicrobial classes were categorized as R2. MDR isolates were defined as those exhibiting resistance to at least one antimicrobial agent in three or more antimicrobial classes.

Statistical analysis

Data were entered and analyzed using Microsoft Excel (Microsoft Corporation, Redmond, WA). Descriptive statistics were used to summarize the data. Categorical variables are presented as frequencies and percentages in tables and figures. Since this was a descriptive study, no inferential statistical tests were performed.

## Results

**Figure 1:**
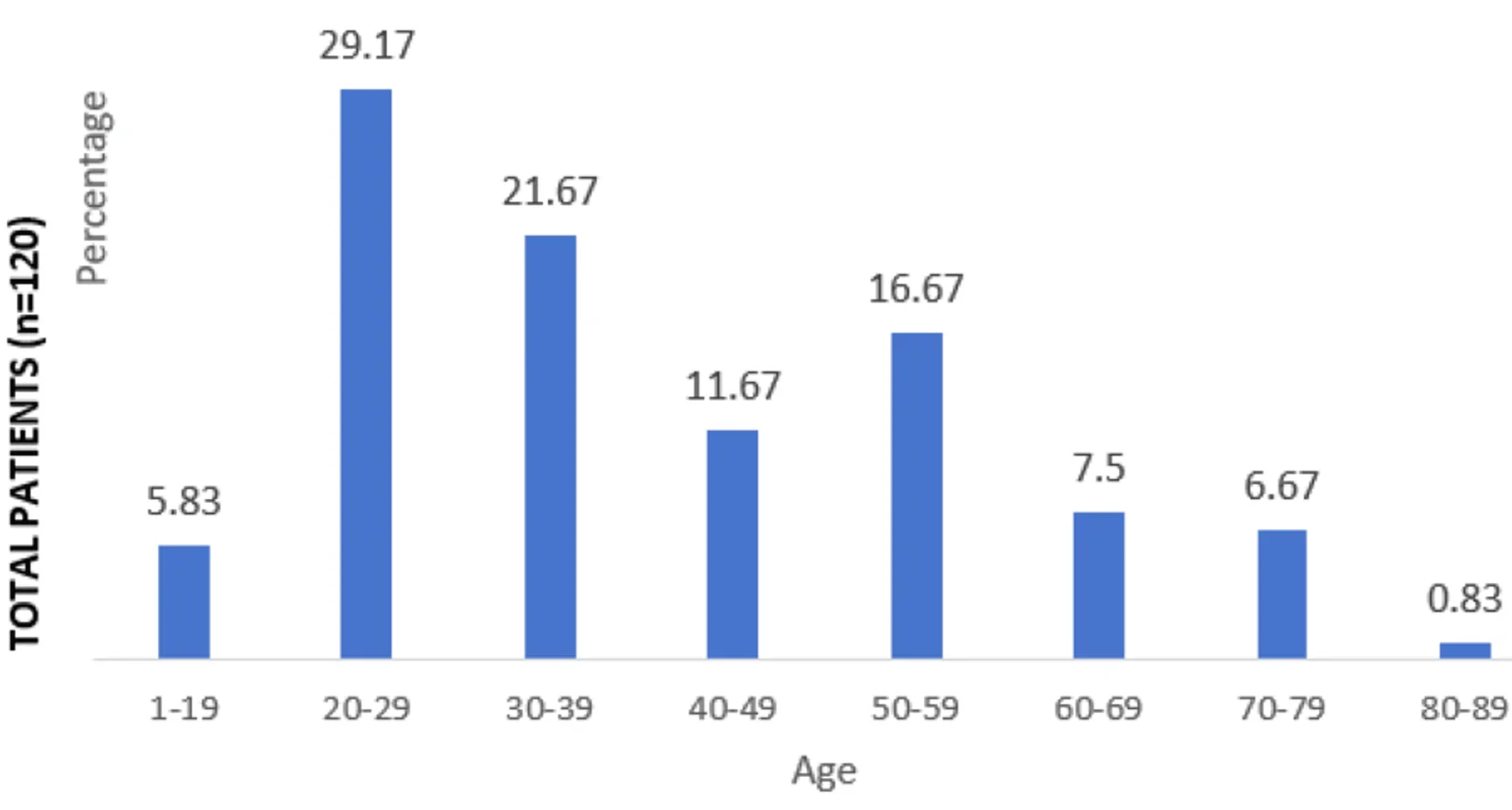
Age demographics of LRTI patients LRTI: lower respiratory tract infection

The study included a total of 120 clinically validated cases of LRTI. The distribution across age groups varied considerably. The lowest prevalence was observed in the 80-89 years age group, which accounted for one (0.83%) case, whereas the highest prevalence was recorded in the 20-29 years age group, with 35 (29.17%) cases. The 30-39 years age group represented 26 (21.67%) cases, while the 50-59 years age group accounted for 20 (16.67%) cases. The 40-49 years age group contributed 14 (11.67%) cases, while the 60-69 years, 70-79 years, and 10-19 years age groups had nine (7.50%), eight (6.67%), and seven (5.83%) cases, respectively. Overall, these findings indicate that LRTIs were more frequently observed among young and middle-aged adults, whereas their occurrence was relatively lower among older adults (Figure 1).

**Figure 2:**
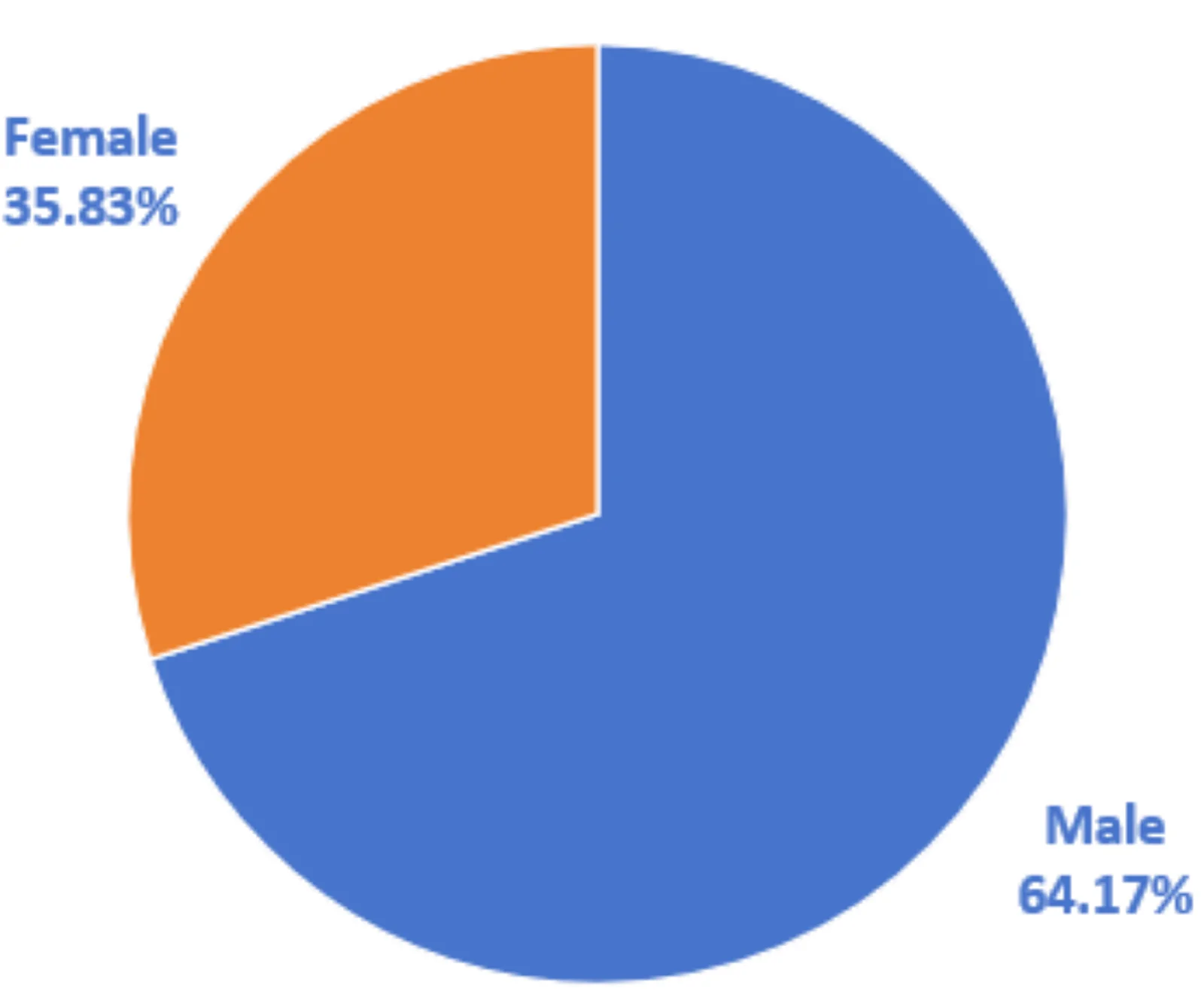
Gender distribution of LRTI cases LRTI: lower respiratory tract infection

Among the 120 clinically confirmed cases of LRTI included in the study, males constituted the majority of patients. Of the total cases, 77 (64.17%) were males, whereas 43 (35.83%) were females. Overall, the findings indicate a higher prevalence of LRTIs among male patients than among female patients during the study period (Figure 2).

**Figure 3:**
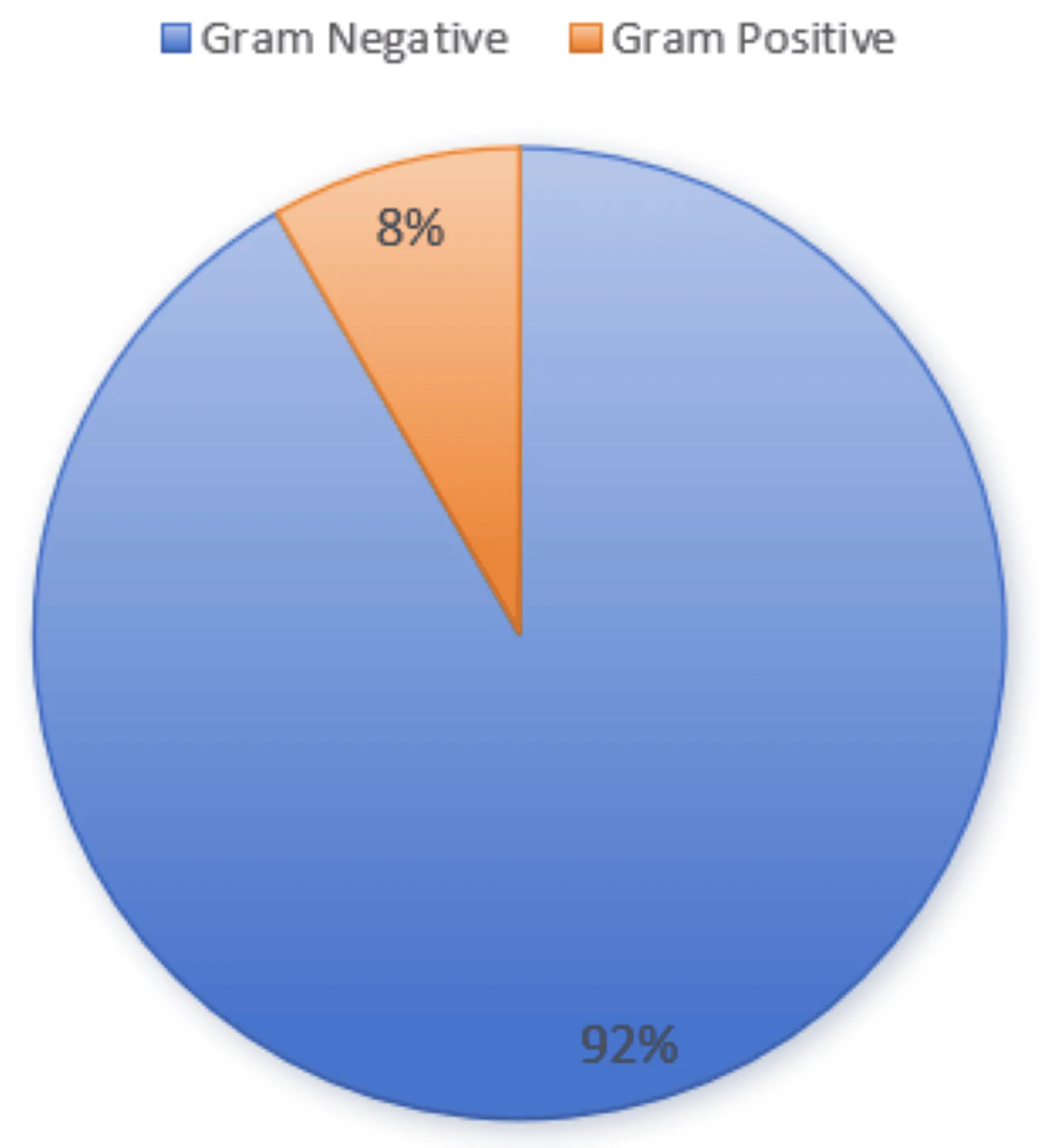
Types of isolates

Among the 120 bacterial isolates obtained from clinically diagnosed LRTI cases, Gram-negative bacteria were the most prevalent, accounting for 110 (91.67%) of the isolates, while Gram-positive bacteria accounted for only 10 (8.33%) of the isolates. These findings indicate that Gram-negative bacteria were the predominant bacterial isolates associated with LRTIs in this study (Figure 3).

**Figure 4:**
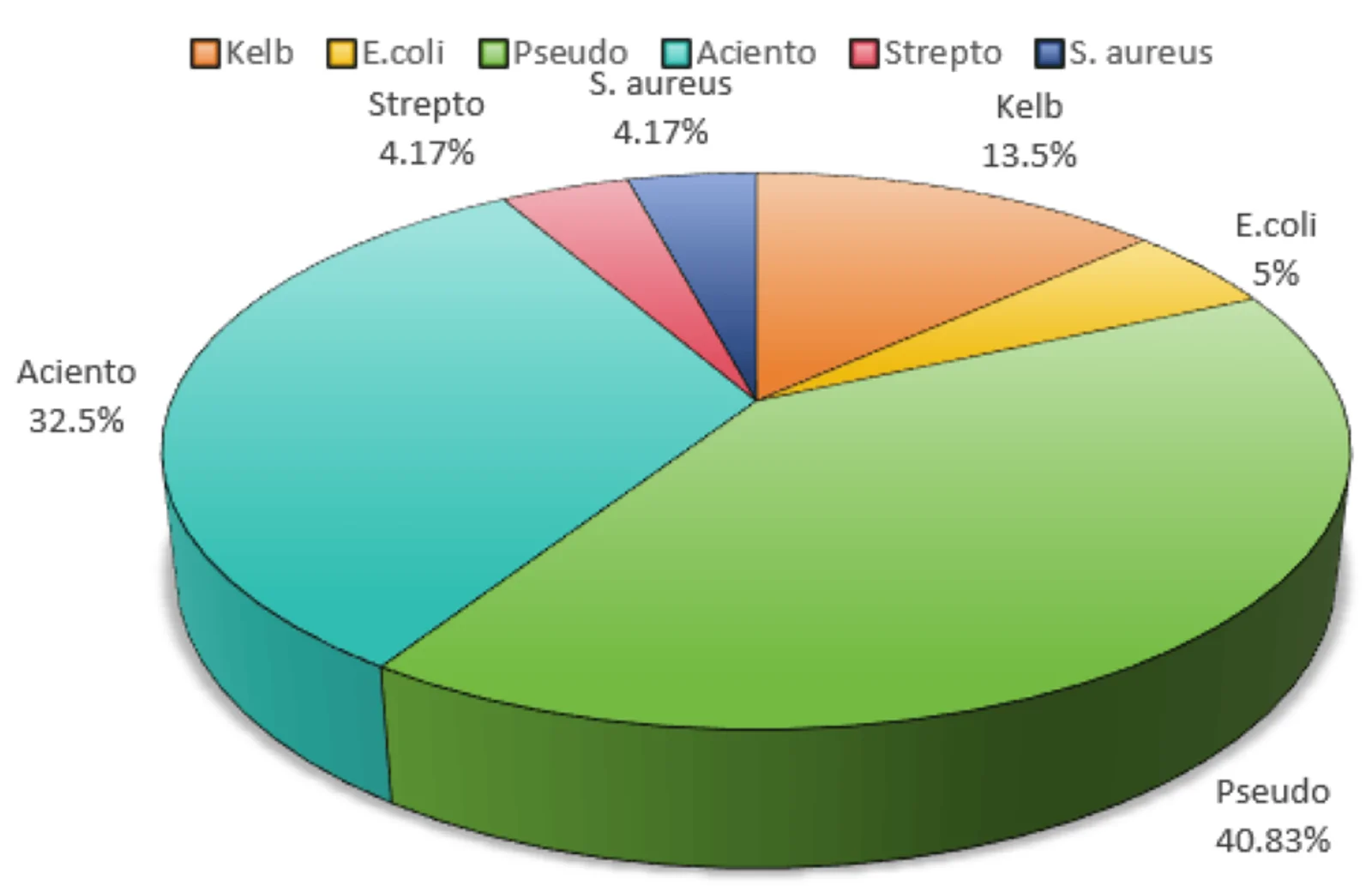
Growth pattern seen in culture-positive samples

Among the 120 culture-positive isolates collected from clinically verified LRTI cases, *Pseudomonas *spp.* *emerged as the most frequently isolated species, comprising 49 (40.83%) isolates, while *Acinetobacter *spp. followed with 39 (32.50%) isolates. *Klebsiella* spp. were recovered from 16 (13.33%) specimens, while *E. coli* was identified in six (5.00%) samples.* Streptococcus *spp. and *Staphylococcus aureus (S. aureus)* were the most infrequent isolates, with each representing five (4.17%) samples. In this study, *Pseudomonas *spp. and* Acinetobacter* spp were the main bacterial pathogens linked to LRTIs (Figure 4).

**Figure 5:**
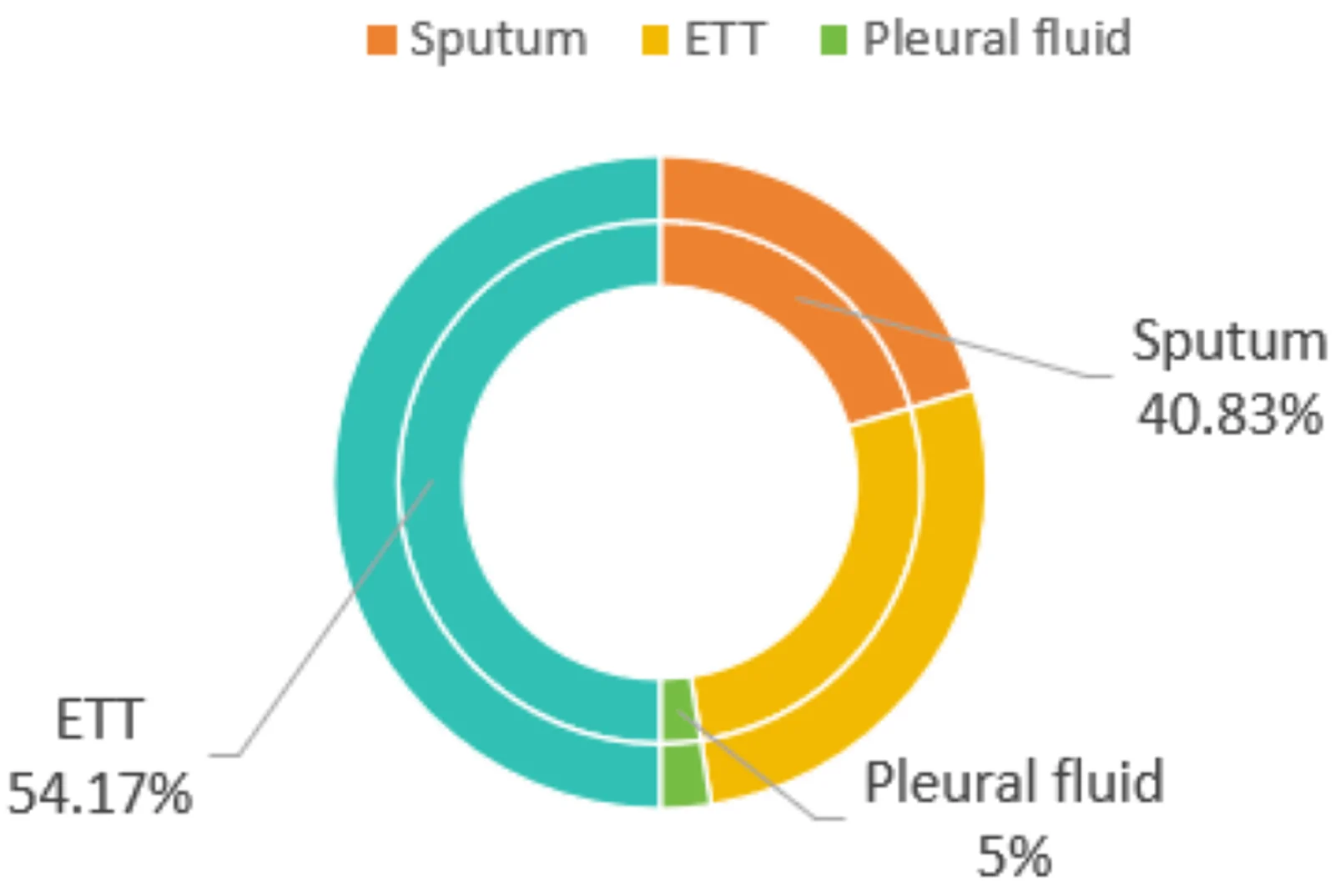
Distribution of LRTI among different clinical specimens LRTI: lower respiratory tract infection

The specimen-wise distribution of isolates varied across the different specimen types. The highest number of isolates was recovered from endotracheal tube (ETT) specimens, accounting for 65 (54.17%) isolates, while sputum specimens contributed 49 (40.83%) isolates. The lowest number of isolates was recovered from pleural fluid samples, accounting for six (5.00%) isolates. Overall, these findings indicate that ETT specimens were the predominant source of bacterial isolates in the present study, whereas pleural fluid specimens yielded the fewest isolates (Figure 5).

Table 1 summarizes the antimicrobial susceptibility profile of the Gram-negative isolates recovered from patients with LRTIs. Among the isolates, *E. coli* showed the greatest susceptibility to amikacin, with 83.33% (5/6) of isolates being sensitive. In contrast, *Klebsiella *spp. displayed generally poor susceptibility, with the highest sensitivity observed for cefepime at 31.25% (5/16). For *P. aeruginosa*, aztreonam demonstrated the best activity, with 48.98% (24/49) of isolates remaining susceptible. Likewise, *Acinetobacter* spp. exhibited maximum susceptibility to doxycycline, to which 38.46% (15/39) of isolates were sensitive.

**Table 1:** Antimicrobial susceptibility pattern of Gram-negative organisms NT: not tested

Antibiotics	*Escherichia coli *(n = 6)		*Klebsiella *spp.(n = 16)		*Pseudomonas aeruginosa *(n = 49)		*Acinetobacter *(n = 39)	
	Sensitive (%)	Resistant (%)	Sensitive (%)	Resistant (%)	Sensitive (%)	Resistant (%)	Sensitive (%)	Resistant (%)
Amoxicillin-clavulanate	66.67	33.33	6.25	93.75	NT	NT	NT	NT
Gentamicin	66.67	33.33	25.00	75	NT	NT	15.38	84.62
Ciprofloxacin	16.67	83.33	6.25	93.75	0.00	100	2.56	89.74
Co-trimoxazole	33.33	66.67	6.25	93.75	NT	NT	17.95	66.67
Cefepime	16.67	83.33	31.25	62.5	0.00	100	0.00	100
Amikacin	83.33	16.67	12.50	87.5	NT	NT	NT	NT
Meropenem	0.00	100	0.00	100	0.00	100	NT	NT
Aztreonam	0.00	100	12.50	87.5	48.98	51.02	NT	NT
Ceftazidime	NT	NT	0.00	100	8.16	91.84	2.56	89.74
Piperacillin	NT	NT	NT	NT	0.00	100	NT	NT
Piperacillin-tazobactam	NT	NT	NT	NT	0.00	100	2.56	89.74
Levofloxacin	NT	NT	NT	NT	0.00	100	NT	NT
Imipenem	NT	NT	NT	NT	0.00	100	2.56	89.74
Doxycycline	NT	NT	NT	NT	NT	NT	38.46	66.67

Overall, resistance to multiple antimicrobial agents was common among the Gram-negative pathogens. Complete resistance to meropenem and aztreonam was observed among *E. coli* isolates. *Klebsiella *spp. showed particularly high resistance to amoxicillin-clavulanate, ciprofloxacin, and co-trimoxazole, with resistance rates reaching 93.75%. A concerning level of resistance was also evident among* P. aeruginosa,* which demonstrated 100% resistance to several routinely used agents, including cefepime, piperacillin, piperacillin-tazobactam, levofloxacin, and imipenem. Similarly, *Acinetobacter *spp. exhibited marked resistance to most tested antibiotics, with complete resistance observed against cefepime and very high resistance rates noted for ciprofloxacin, ceftazidime, piperacillin-tazobactam, and imipenem. These findings highlight the substantial burden of AMR among Gram-negative respiratory pathogens in the study population Table 1.

Table 2 outlines how antimicrobial susceptibility and resistance were distributed among the 10 Gram-positive pathogens isolated during our study (n = 5 for each species). Within this small cohort, *Staphylococcus aureus* displayed extensive resistance to most conventional frontline options, yet remained fully vulnerable to key reserve treatments. Notably, the pathogen showed 100% susceptibility to linezolid, followed by a moderate sensitivity rate of 60% for both ofloxacin and cefotaxime. In contrast, standard options performed poorly: only 40% of the isolates were sensitive to tetracycline, and susceptibility plunged to a critical 20% for penicillin, erythromycin, clindamycin, and cefoxitin.

**Table 2:** Antimicrobial susceptibility pattern of Gram-positive organisms NT: not tested

Antibiotics	*Staphylococcus aureus* (n = 5)		*Streptococcus pyogenes* (n = 5)	
					Sensitive (%)	Resistant (%)	Sensitive (%)	Resistant (%)
	Ofloxacin	60	40	NT	NT
Penicillin	20	80	80	20
Cefotaxime	60	40	NT	NT
Erythromycin	20	80	40	60
Clindamycin	20	80	60	40
Tetracycline	40	60	40	60
Linezolid	100	0	60	40
Cefoxitin	20	80	60	40
Vancomycin	NT	NT	100	0

On the other hand, *Streptococcus pyogenes (S. pyogenes) *retained a favorable susceptibility profile toward core therapeutic choices, with every single isolate (100%) demonstrating complete vulnerability to vancomycin. We also observed strong susceptibility to penicillin (80%), alongside a 60% sensitivity rate for clindamycin, cefoxitin, and linezolid (60% sensitive / 40% resistant). The most pronounced resistance features for *S. pyogenes* were restricted to erythromycin and tetracycline, with resistance rates peaking at 60% for both antimicrobial agents Table 2.

**Figure 6:**
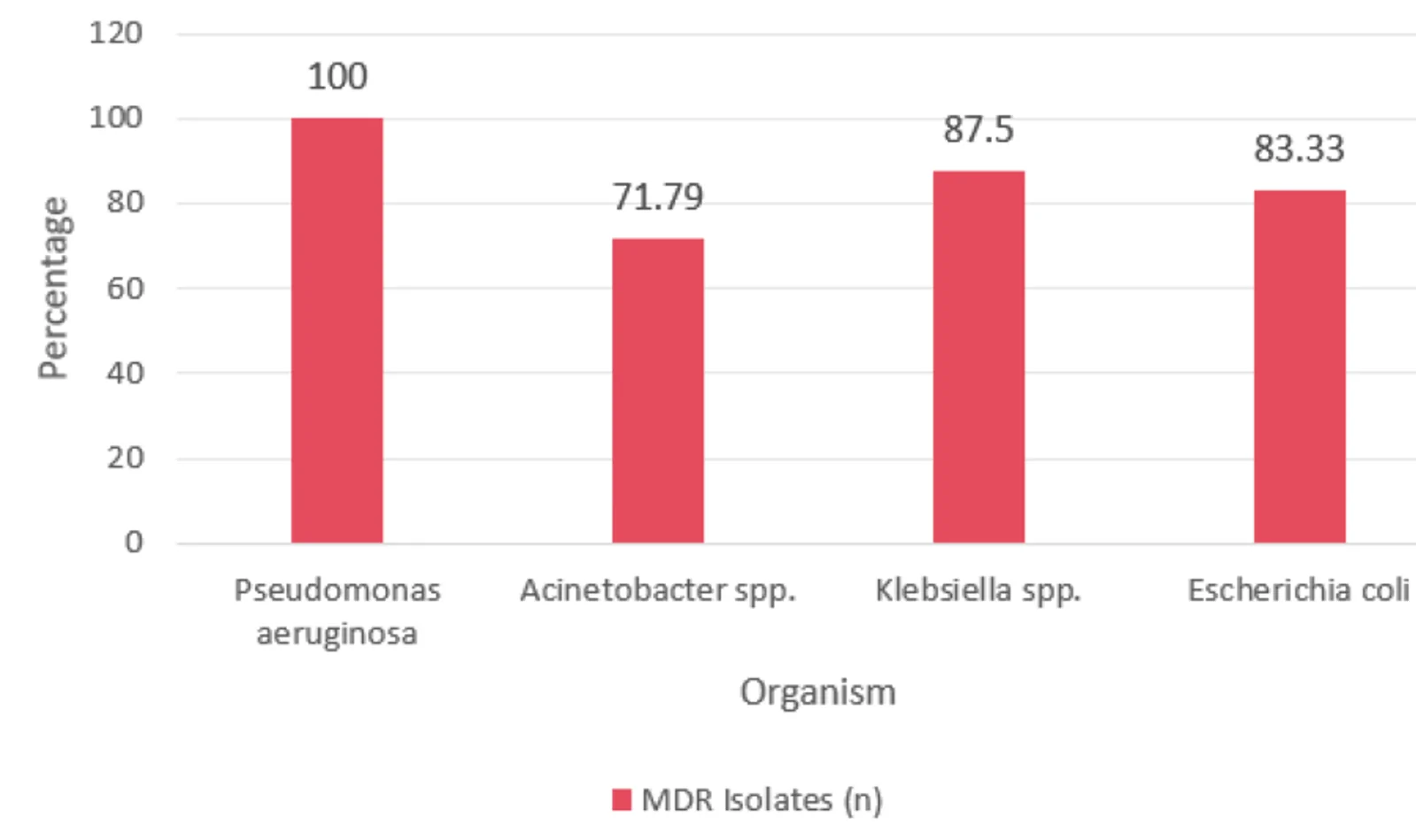
Organism-wise distribution of Gram-negative MDR isolates MDR: multidrug-resistant

Among the 110 Gram-negative bacterial isolates recovered in this study, an overwhelming majority of 96 (87.27%) were identified as MDR. As shown in Figure 6, species-specific evaluation revealed alarming resistance profiles, led by *P. aeruginosa* with an absolute MDR rate of 100% (49/49). High frequencies of multidrug resistance were also prominent among the remaining pathogens, with *Klebsiella *spp. at 87.50% (14/16) and *E. coli* at 83.33% (5/6), followed by *Acinetobacter *spp. at 71.79% (28/39). Collectively, these distributions highlight that opportunistic, non-fermenting Gram-negative bacilli constitute the principal MDR threats associated with LRTIs in the study (Figure 6).

## Discussion

LRTIs continue to be among the major causes of illness and death globally, significantly impacting the healthcare system for all age demographics. Gram-negative bacteria are increasingly acknowledged as significant causal factors in pneumonia, whether acquired in the community or within hospitals, especially among hospitalized and ventilated individuals, where MDR poses a rising global risk [12,13]. The rising occurrence of MDR Gram-negative bacilli has greatly complicated empirical treatment and is linked to unfavorable clinical outcomes [14].

In the present study, the highest proportion of LRTI cases was observed among individuals aged 20-29 years, followed by those aged 30-39 years. Similar age-related trends have been reported in previous studies, where young and middle-aged adults constituted a substantial proportion of respiratory infection cases. Increased occupational exposure, environmental factors, and greater social interaction may contribute to the higher occurrence of LRTIs in these age groups. [15]. This study revealed a predominance of males. This is consistent with previous research demonstrating that men are more susceptible to respiratory infections because of elements like smoking, alcohol intake, workplace exposure, and greater healthcare interactions [15,16]. Differences in immune responses based on gender may further lead to heightened susceptibility in males.

In this particular study, Gram-negative bacteria were the main bacterial isolates, with *Pseudomonas *spp.*, Acinetobacter *spp.*, Klebsiella *spp.*,* and* E. coli *being the most commonly identified pathogens. This aligns with worldwide reports showing that Gram-negative bacilli, especially non-fermenting species like *P. aeruginosa* and *Acinetobacter baumannii (A. baumannii),* are significant contributors to pneumonia acquired in hospitals and infections related to ventilators [13,17]. These organisms possess intrinsic resistance mechanisms and can persist in healthcare environments, characteristics that have been associated with their ability to survive and disseminate in healthcare settings [17]. The dominance of *Pseudomonas* spp. and *Acinetobacter* spp. in this investigation carries clinical significance, as these organisms are known to form biofilms on respiratory equipment such as endotracheal tubes. Biofilm formation may contribute to persistent colonization and has been associated with ventilator-associated pneumonia (VAP) in previous studies [16,18]. However, VAP was not specifically evaluated in the present study [16,18].

Distribution by specimen indicated that ETT samples produced the largest number of isolates, with sputum and pleural fluid samples following. The predominance of endotracheal tube ETT specimens reflects the substantial representation of mechanically ventilated patients in the study population. Prolonged intubation has been associated with respiratory tract colonization and infection with MDR organisms in previous studies, potentially through disruption of normal airway defenses and biofilm formation on artificial surfaces [16]. However, the present study did not specifically assess the duration of mechanical ventilation or its association with MDR infection. AST demonstrated variable sensitivity patterns among Gram-negative pathogens.* E. coli* exhibited the highest susceptibility to amikacin, whereas *Klebsiella *spp. showed limited susceptibility even to its most active agent, cefepime. *P. aeruginosa* demonstrated moderate susceptibility to aztreonam, while *Acinetobacter *spp. were comparatively more susceptible to doxycycline. Despite the observed activity of these agents, overall susceptibility remained low among most Gram-negative isolates.

Furthermore, high levels of resistance were observed against several commonly used antibiotics, particularly cephalosporins, fluoroquinolones, and carbapenems. This pattern is consistent with global reports describing increasing antimicrobial resistance among Gram-negative bacilli, largely driven by the production of extended-spectrum β-lactamases (ESBLs) and carbapenemases [14,18]. The emergence of carbapenem-resistant organisms is especially concerning because it significantly limits available treatment options and increases reliance on last-resort antimicrobial agents.

Significantly, out of the 110 Gram-negative clinical isolates, an overwhelming majority of 96 (87.27%) met the criteria for MDR. The predominant burden of these MDR phenotypes was represented by Pseudomonas aeruginosa and *Acinetobacter *spp.*,* followed progressively by *Klebsiella *spp. and *E. coli.* These observations closely mirror several ICU-centric studies where non-fermenting Gram-negative bacilli emerge as primary MDR vectors owing to their intrinsic resistance architecture and a pronounced capacity to acquire additional resistance determinants via horizontal gene transfer mechanisms [14, 17]. The high proportion of MDR organisms observed in this study may reflect complex clinical and healthcare-associated factors reported in previous literature; however, these factors were not directly evaluated in the present study. Previous studies have identified inappropriate antimicrobial use and healthcare-associated exposures as potential contributors to the development and dissemination of MDR organisms [14,18].

Interestingly, while the Gram-negative pathogens in this study displayed severe resistance profiles, our Gram-positive isolates remained responsive to critical reserve antimicrobials. Most notably, *S. aureus *exhibited 100% susceptibility to linezolid, while *S. pyogenes *demonstrated complete vulnerability to vancomycin. Furthermore, *S. pyogenes* maintained a moderate sensitivity rate of 60% to linezolid. These findings track closely with broader epidemiological data showing that these specific protein-synthesis inhibitors and glycopeptides continue to hold reliable therapeutic efficacy against Gram-positive respiratory pathogens. Conversely, the low susceptibility rates observed for traditional front-line options like penicillin and erythromycin in our *S. aureus* isolates highlight the clear necessity of managing these cornerstone agents carefully to prevent further resistance decay in Gram-positive strains [4].

The results of this investigation indicate a substantial burden of Gram-negative bacterial infections and MDR pathogens among patients with lower respiratory tract infections. These findings emphasize the importance of rapid microbiological diagnosis, routine antimicrobial susceptibility testing, implementation of antimicrobial stewardship programs, and strict infection prevention and control measures to reduce the emergence and transmission of MDR pathogens and improve patient outcomes.

Limitations

This study has certain limitations. The study was conducted at a single tertiary-care center, which may limit the generalizability of the findings to other healthcare settings. The relatively limited sample size and predominance of endotracheal tube specimens may also restrict extrapolation of the findings to the broader LRTI population. Detailed patient-level clinical risk factors and outcomes, such as mortality and length of hospital stay, were not evaluated. Furthermore, microbiological findings alone may not always allow complete differentiation between respiratory tract colonization and true infection. Molecular characterization of resistance mechanisms was also not performed. Multicenter studies incorporating detailed clinical, epidemiological, and molecular analyses are needed to validate and extend these findings.

## Conclusions

The current research revealed a dominance of Gram-negative bacterial pathogens in LRTIs, including *Pseudomonas *spp. and* Acinetobacter *spp. as the most prevalent isolates recovered. A high prevalence of MDR Gram-negative bacteria was observed, highlighting the growing challenge posed by AMR in the management of LRTIs. AST showed different susceptibility patterns among the isolates, highlighting the necessity of regular culture and susceptibility testing for choosing suitable antibiotic treatment. Ongoing monitoring of bacterial pathogens, treatment guided by AST, efficient antimicrobial management, and strict infection-control protocols are vital to enhance patient outcomes and inhibit the dissemination of resistant strains.
